# Pyruvate anaplerosis is a mechanism of resistance to pharmacological glutaminase inhibition in triple-receptor negative breast cancer

**DOI:** 10.1186/s12885-020-06885-3

**Published:** 2020-05-25

**Authors:** Dean C. Singleton, Anne-Lise Dechaume, Pamela M. Murray, William P. Katt, Bruce C. Baguley, Euphemia Y. Leung

**Affiliations:** 1 Auckland Cancer Society Research Centre, School of Medical Sciences, Faculty of Medical and Health Sciences University of Auckland, Private Bag, Auckland, 92019 New Zealand; 2 Maurice Wilkins Centre for Molecular Biodiscovery, University of Auckland, Symonds Street, Auckland, 1010 New Zealand; 3 Department of Molecular Medicine, Cornell University, Ithaca, New York, 14853 USA

**Keywords:** Glutaminolysis, Glutamine, Pyruvate, Triple-receptor negative breast Cancer, Cancer metabolism, Glutaminase inhibitor

## Abstract

**Background:**

Glutamine serves as an important nutrient with many cancer types displaying glutamine dependence. Following cellular uptake glutamine is converted to glutamate in a reaction catalysed by mitochondrial glutaminase. This glutamate has many uses, including acting as an anaplerotic substrate (via alpha-ketoglutarate) to replenish TCA cycle intermediates. CB-839 is a potent, selective, orally bioavailable inhibitor of glutaminase that has activity in Triple receptor-Negative Breast Cancer (TNBC) cell lines and evidence of efficacy in advanced TNBC patients.

**Methods:**

A panel of eleven breast cancer cell lines was used to investigate the anti-proliferative effects of the glutaminase inhibitors CB-839 and BPTES in different types of culture medium, with or without additional pyruvate supplementation. The abundance of the TCA cycle intermediate fumarate was quantified as a measure if TCA cycle anaplerosis. Pyruvate secretion by TNBC cultures was then assessed with or without AZD3965, a monocarboxylate transporter 1 (MCT1) inhibitor. Finally, two dimensional (2D) monolayer and three dimensional (3D) spheroid assays were used to compare the effect of microenvironmental growth conditions on CB-839 activity.

**Results:**

The anti-proliferative activity of CB-839 in a panel of breast cancer cell lines was similar to published reports, but with a major caveat; growth inhibition by CB-839 was strongly attenuated in culture medium containing pyruvate. This pyruvate-dependent attenuation was also observed with a related glutaminase inhibitor, BPTES. Studies demonstrated that exogenous pyruvate acted as an anaplerotic substrate preventing the decrease of fumarate in CB-839-treated conditions. Furthermore, endogenously produced pyruvate secreted by TNBC cell lines was able to act in a paracrine manner to significantly decrease the sensitivity of recipient cells to glutaminase inhibition. Suppression of pyruvate secretion using the MCT1 inhibitor AZD3965, antagonised this paracrine effect and increased CB-839 activity. Finally, CB-839 activity was significantly compromised in 3D compared with 2D TNBC culture models, suggesting that 3D microenvironmental features impair glutaminase inhibitor responsiveness.

**Conclusion:**

This study highlights the potential influence that both circulating and tumour-derived pyruvate can have on glutaminase inhibitor efficacy. Furthermore, it highlights the benefits of 3D spheroid cultures to model the features of the tumour microenvironment and improve the in vitro investigation of cancer metabolism-targeted therapeutics.

## Background

Cancer cells utilise glutamine to aid in the biosynthetic, bioenergetic and redox needs that are associated with proliferation [1–4]. Many Triple-receptor Negative Breast Cancer (TNBC) cell lines are particularly dependent on glutamine for growth and viability [5, 6]. These cells acquire glutamine and then convert it to glutamate in a reaction catalysed by mitochondrial glutaminase – predominantly the *GAC* splice variant encoded by the *GLS* gene [5, 6]. The glutamate derived from glutamine has many uses, including glutathione synthesis or further metabolism to α-ketoglutarate (αKG) by glutamate dehydrogenase/aminotransferase-catalysed reactions [1, 4]. This αKG contributes to numerous biosynthetic and epigenetic processes or can act as an anaplerotic substrate to replenish tricarboxylic acid (TCA) cycle metabolites that have been exported from the mitochondria for the production of biomass [3]. Once αKG enters the TCA cycle it can support TCA cycle flux either through oxidative decarboxylation or reductive carboxylation [7–11].

This dependence on glutamine anaplerosis renders TNBC cells at increased sensitivity to pharmacological glutaminase inhibition both in vitro and in vivo [6, 12, 13]. Clinically, glutaminase inhibition is emerging as a promising therapeutic avenue for the management of advanced TNBC. CB-839 (Telaglenastat) is a potent, selective, orally bioavailable first-in-class glutaminase inhibitor that has demonstrated promise in the management of metastatic TNBC in Phase I/II studies [14, 15]. When combined with paclitaxel, CB-839 was well tolerated with evidence of antitumour activity in heavily pre-treated patients. Yet, although an objective response rate (ORR) of 22% was observed in the Phase I study (doses ≥600 mg BID, *n* = 27), the outcome of the Phase II study was less encouraging with ORR of 6% (800 mg BID, *n* = 16, “Third Line +” cohort) [14, 15]. A greater mechanistic understanding of the pharmacology of glutaminase inhibition, development of rational drug combinations and the identification and validation of biomarkers may assist in further clinical development of glutaminase inhibitors for TNBC treatment.

While preclinical and clinical studies have confirmed the sensitivity of TNBC to glutaminase inhibition, additional reports in a variety of cancer types have uncovered a set of intrinsic and extrinsic determinants that can impair cellular sensitivity to glutaminase inhibitors. Cells derived from mouse models of non-small cell lung cancer (NSCLC) were highly dependent on glutamine for TCA cycle anaplerosis and proliferation when grown in cell culture but utilised minimal glutamine when grown in vivo, relying instead on glucose metabolism to fuel the TCA cycle [16]. This finding along with results from clinical in vivo and ex vivo isotope tracer studies suggest that the tumour microenvironment has a strong influence on cellular metabolic programmes and the potential to influence the efficacy of metabolism-targeted therapies [17, 18].

One possible contributor for the loss of glutamine dependence observed in vivo is the lower cystine concentration in tumours compared with cell culture medium. Growing cells in vitro in physiological concentrations of cystine (20–50 μM) suppressed the level of glutamine anaplerosis and subsequently desensitised cells to CB-839 [19]. On the contrary, administering cystine to mice increased plasma cystine levels and promoted glutamine anaplerotic flux in subcutaneous tumour xenografts [19]. An additional, intrinsic cellular characteristic is required for this effect; the expression of the glutamate/cystine antiporter (xCT) subunit *SLC7A11* promotes this cystine-dependent increase in glutamine utilisation resulting in glutamine dependence [19]. Many oncogenic processes promote the elevated expression of *SLC7A11*, including *KEAP1* mutation and the subsequent NRF2 (*NFE2L2*)-driven antioxidant response [20].

A number of other metabolites have been identified that can reduce glutamine dependence. For example, increasing the levels of exogenous glutamate can support cell proliferation in times of glutamine deprivation or glutaminase inhibition [20–23]. Likewise, addition of pyruvate or oxaloacetate could prevent apoptosis during acute glutamine deprivation but was unable to support cell proliferation [24]. Addition of extracellular deoxynucleosides was also shown to render TNBC cells resistant to glutamine deprivation [25]. Yet, whether these extrinsic factors contribute to the decrease in glutamine metabolism observed in many tumours compared with in vitro conditions and the potential impact on antitumour activity of glutaminase inhibitors is unknown.

In this study we investigated a key difference in culture medium composition that can influence the sensitivity of TNBC cell lines to pharmacological glutaminase inhibition. We show that extracellular pyruvate, at physiological concentrations of 20–100 μM, can significantly impair CB-839 potency in vitro by acting as an anaplerotic substrate. Normal blood pyruvate concentration is reported in the range of 30–150 μM [26–28]. Furthermore, we demonstrate that paracrine secretion of de novo produced pyruvate into the extracellular environment can act as a source of pyruvate and this process can be antagonised using a monocarboxylate transporter 1 (MCT1) inhibitor. Our work highlights the potential for both systemic- and paracrine tumour-derived pyruvate to limit the antitumour activity of glutaminase inhibitors and uncovers a possible rational combination that includes addition of MCT1 inhibitor to glutaminase inhibitor therapy.

## Methods

### Mammalian cell culture

Cell lines used in this study were sourced from American Type Culture Collection (Manassas, VA), with the exception of Hs578T (The European Collection of Authenticated Cell Cultures, Salisbury, UK), MDA-MB-231-luc-D3H2LN (Caliper Life Sciences, Hopkinton, MA, acquired January 2008) and SUM159PT (Asterand Bioscience, Detroit, MI, acquired January 2010). All cell lines were tested negative for mycoplasma contamination (PlasmoTest™ - Mycoplasma Detection kit, InvivoGen, San Diego, CA), but have not been STR authenticated since 2013. Cells were cultured in either RPMI 1640 (11,875,093, ThermoFisher Scientific, Auckland, New Zealand), αMEM (12,000,063, ThermoFisher Scientific) or DMEM (10,569,010, ThermoFisher Scientific) supplemented with 5% v/v foetal bovine serum (FBS; Moregate Biotech, Hamilton, New Zealand), as indicated. SUM159PT was routinely cultured in DMEM:F12 (1:1, ThermoFisher Scientific) + 5% FBS + 1 μg/mL hydrocortisone (Sigma-Aldrich, Auckland, New Zealand) + 5 μg/mL insulin (Sigma-Aldrich).

### Chemicals

CB-839 (S7655, Selleck Chemicals, Houston, TX), Bis-2-(5-phenylacetamido-1,3,4-thiadiazol-2-yl) ethyl sulphide (BPTES) (19,284, Cayman Chemical, Ann Arbor, MI) and AZD3965 (S7339, Selleck Chemicals) were dissolved in DMSO to generate 10–25 mM stock solutions, aliquoted and stored at − 20 °C. Sterile 100 mM sodium pyruvate was acquired from ThermoFisher Scientific.

### ^3^H-thymidine incorporation assay

Proliferation was measured using a thymidine incorporation assay, as described previously [29]. Briefly, cells were seeded (1–3 × 10^3^ per well) in 96-well plates in the presence of varying concentrations of inhibitor for 3 or 4 days, as indicated. ^3^H-thymidine (0.04 μCi per well for two dimensional (2D) monolayer culture or 0.08 μCi per well for three dimensional (3D) spheroid culture, see below) was added (6 h for 2D monolayer culture or 16 h for 3D spheroid culture) prior to harvest, cells were then harvested on glass fiber filters using an automated TomTec harvester. Filters were incubated with Betaplate Scint and thymidine incorporation measured in a Trilux/Betaplate counter. Effects of inhibitors on the incorporation of ^3^H-thymidine into DNA were determined relative to the control (non-drug-treated) samples.

### Fumarate assay

2.5 × 10^5^ MDA-MB-231 cells were seeded per well of 6-well plates (140,675, ThermoFisher Scientific) in 1 mL of RPMI 1640 + 5% FBS. An additional 1 mL of RPMI 1640 + 5% FBS was added containing either CB-839 to a final concentration of 1 μM or an equivalent volume of DMSO. 20 μL of 100 mM sodium pyruvate (or an equivalent volume of 18.2 MΩ·cm at 25 °C “Milli-Q” water) was added to achieve a final concentration of 1 mM. After 24 h incubation culture medium was aspirated and cell monolayers were washed twice in cold saline and then lysed on ice using 0.1 mL of Fumarate Assay Buffer (Fumarate Assay Kit ab102516, Abcam plc). Lysate was transferred into 1.5 mL microcentrfuge tubes and samples were then centrifuged for 10 min at 13,000×g at 4 °C to remove debris. The assay was conducted as detailed in the instruction booklet using 50 μL of the lysate supernatant. Sample absorbance at 450 nm was measured in an EnSpire Multimode Plate Reader (PerkinElmer) and fumarate amount (nmol) was interpolated from the standard curve. Lysate protein concentration was then quantified using bicinchoninic acid assay. The amount of fumarate (nmol) was then normalised to either initial number of cells seeded or protein (mg) to account for changes in cell number caused by drug treatment.

### Pyruvate assay

Hs578T (1.5 × 10^5^), MDA-MB-231-luc-D3H2LN (2.5 × 10^5^) or SUM159PT (2.5 × 10^5^) cells were seeded per well of 6-well plates in 1 mL of RPMI 1640 + 5% FBS. An additional 1 mL of RPMI 1640 + 5% FBS was added containing either AZD3965 to a final concentration of 1 μM or an equivalent volume of DMSO. After 48 h incubation, the conditioned culture medium was collected and centrifuged at 800×g to pellet floating cells and cell debris. Aliquots of the conditioned culture medium were diluted 1:3 in Pyruvate Assay Buffer (Pyruvate Assay Kit ab65342, Abcam plc) and pyruvate concentrations were quantified according to the instruction booklet. Briefly, fluorometric signal at excitation/emission 540/590 nm was measured in an EnSpire Multimode Plate Reader and pyruvate concentration was extrapolated from the standard curve.

### Effect of secreted pyruvate on anti-proliferative effect of CB-839

The conditioned culture medium (or unconditioned RPMI 1640 + 5% FBS) described in the pyruvate assay method above was then supplemented with CB-839 to a final concentration of 0.01, 0.1, 1 or 10 μM. MDA-MB-231 cells were then grown in this conditioned culture medium and thymidine incorporation was assessed after 3 days growth.

### 3D spheroid cell culture

On day 0, 5 × 10^3^ cells/well were seeded into ultra-low attachment round bottom 96-well plates (7007, Corning, Kennebunk, ME) in ice-cold 50 μL RPMI 1640 + 5% FBS containing 2.5% (v/v) Geltrex LDEV-Free Reduced Growth Factor Basement Membrane Matrix (A1413202, ThermoFisher Scientific). The plates were centrifuged at 800×g for 10 min and placed in the cell culture incubator to allow establishment of spheroids. On day 1, 50 μL of fresh RPMI 1640 + 5% FBS was added to the plates. On day 4, 100 μL of RPMI 1640 + 5% FBS containing CB-839 at 2× the desired final concentration was added to the spheroid plates. Images of the spheroids were captured using a 10× objective on a JuLI™ Stage Real-Time Cell History Recorder (NanoEnTek, Seoul, Korea) on day 4 and day 8. On day 7 ^3^H-thymidine was added to the cultures and thymidine incorporation was detected following a 16 h overnight incubation.

### Statistical analysis and graphing

Statistical analysis and graphing was conducted using Prism (version 8.0.2, GraphPad Software, Inc.). Comparison of two groups was done by unpaired t test. Comparison of three or more groups was done by one-way or two-way analysis of variance (ANOVA) with Dunnett’s or Tukey’s post hoc test, respectively. Correlation analysis was done using Pearson r correlation coefficient. Values of *P* < 0.05 were considered to be statistically significant. All statistical analyses presented represent data from independent studies (*n* ≥ 2, as indicated).

## Results

### Breast cancer cell lines display differences in sensitivity to pharmacological glutaminase inhibition depending on culture medium composition

**Fig. 1:**
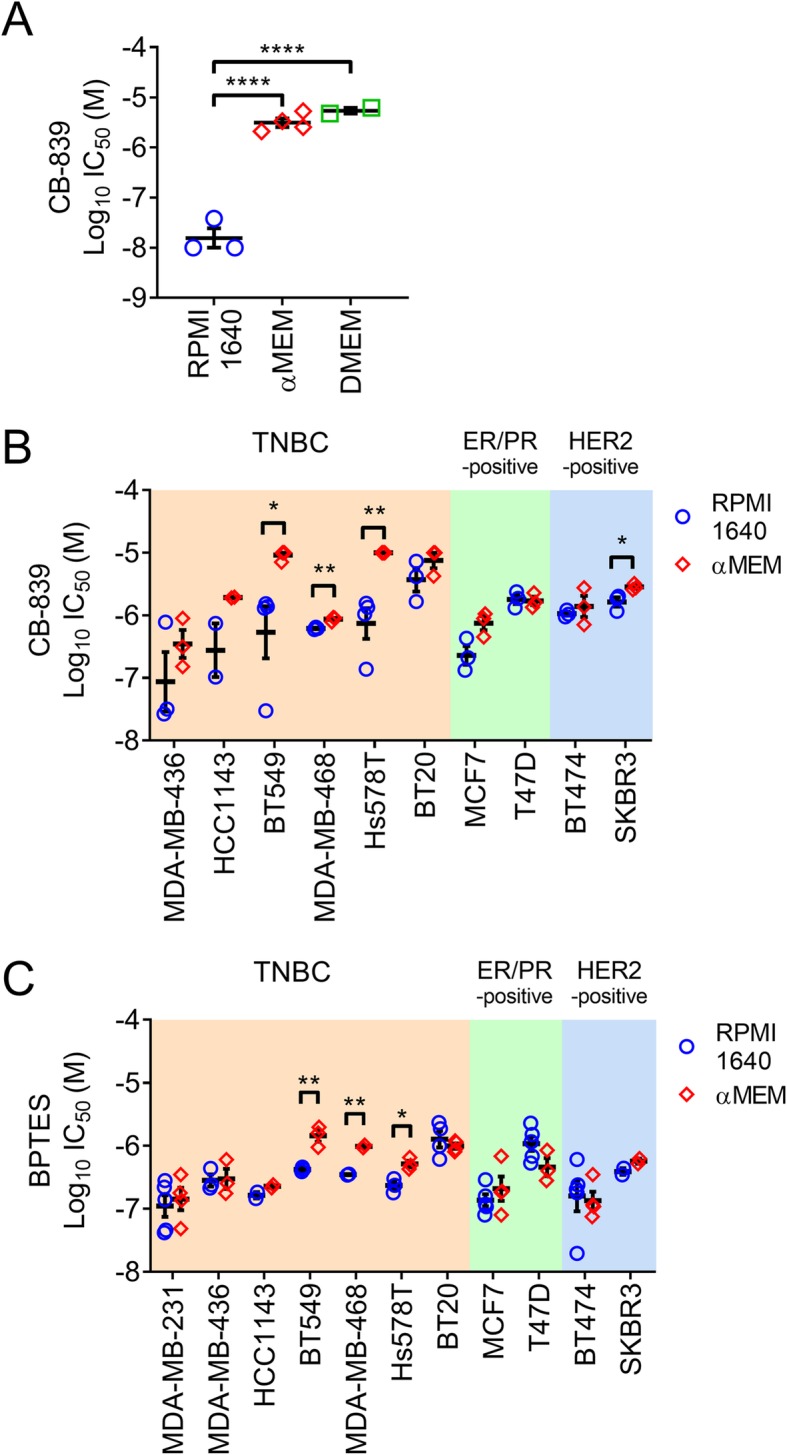
Breast cancer cell lines display differences in sensitivity to pharmacological glutaminase inhibition depending on culture medium composition. **a** MDA-MB-231 cells were more sensitive to 3 days CB-839 exposure when assayed in RPMI 1640 + 5% FBS (RPMI 1640) compared with αMEM + 5% FBS (αMEM) or DMEM + 5% FBS (DMEM) (mean ± SEM, *n* = 2–4, one-way ANOVA). CB-839 (**b**) or BPTES (**c**) display more potent IC_50_ values when assayed in RPMI 1640 + 5% FBS compared with αMEM + 5% FBS in many breast cancer cell lines (mean ± SEM, n = 2–4, unpaired t test)

MDA-MB-231 cells grown in αMEM + 5% FBS (hereafter αMEM) were treated with CB-839 and drug sensitivity was assessed 3 days later by IC_50_ analysis. The observed IC_50_ of 3.3 ± 0.71 μM (mean ± sem, *n* = 4) was > 100-fold higher than the reported value of 26 nM [6] (Fig. 1a), albeit using a different endpoint method (^3^H-thymidine incorporation versus Promega Cell Titer Glo assay). To understand this discrepancy we treated MDA-MB-231 cultures with CB-839 in RPMI 1640 + 5% FBS (hereafter RPMI 1640) as used by Gross et al., 2014 and this change in culture medium resulted in an IC_50_ of 19.3 ± 9.3 nM (*n* = 3), in strong agreement with the published value (Fig. 1a). MDA-MB-231 cultures were also grown and treated with CB-839 in DMEM + 5% FBS (hereafter DMEM) and in this case were also relatively insensitive to CB-839 with an IC_50_ of 5.5 ± 0.71 μM (*n* = 2) (Fig. 1a). These findings suggest that the culture medium composition significantly influences the potency of CB-839.

To explore this relationship further, a panel of 10 additional breast cancer cell lines were treated with CB-839 in either RPMI 1640 or αMEM (Fig. 1b). In general, the TNBC cell lines were more sensitive than the receptor-positive cell lines, with the exception of MCF7, in agreement with published reports [6]. CB-839 IC_50_ values were lower in RPMI 1640 compared with αMEM culture medium in many cell lines tested, with statistically significant effects observed in four cases; BT549, MDA-MB-468, Hs578T and SKBR3, and a trend toward an effect in HCC1143 and MCF7. T47D and BT474 displayed no difference in CB-839 IC_50_ when cultured in either RPMI 1640 or αMEM.

BPTES, a related allosteric glutaminase inhibitor, was then investigated using the same 11 cell line panel (Fig. 1c). In agreement with the CB-839 observations, BPTES IC_50_ values were generally lower in RPMI 1640 compared with αMEM culture medium, with statistically significantly lower values observed in BT549, MDA-MB-468 and Hs578T.

### Extracellular pyruvate concentrations influence CB-839 potency

The import and metabolism of extracellular glutamine serves as a key TCA cycle anaplerotic substrate in proliferating cancer cells. Previous reports have highlighted the potential of oxaloacetate, pyruvate, glutamate or cell permeable α-KG (dimethyl α-KG) to act as alternative anaplerotic substrates that can rescue cell viability during glutamine deprivation and in some cases antagonise the activity of glutaminase inhibitors [25]. We reviewed the composition of the RPMI 1640, αMEM and DMEM formulations used in our studies to identify different components that may be responsible for the difference in sensitivity observed (Supplementary Table S1). Of the numerous different components, one of the key differences noted was sodium pyruvate. While αMEM and DMEM contain 1 mM sodium pyruvate, RPMI 1640 does not contain added pyruvate. We hypothesised that utilisation of extracellular pyruvate by TNBC cells may support mitochondrial anaplerosis, resulting in decreased dependence on glutamine and rendering cells less sensitive to glutaminase inhibition.

**Fig. 2:**
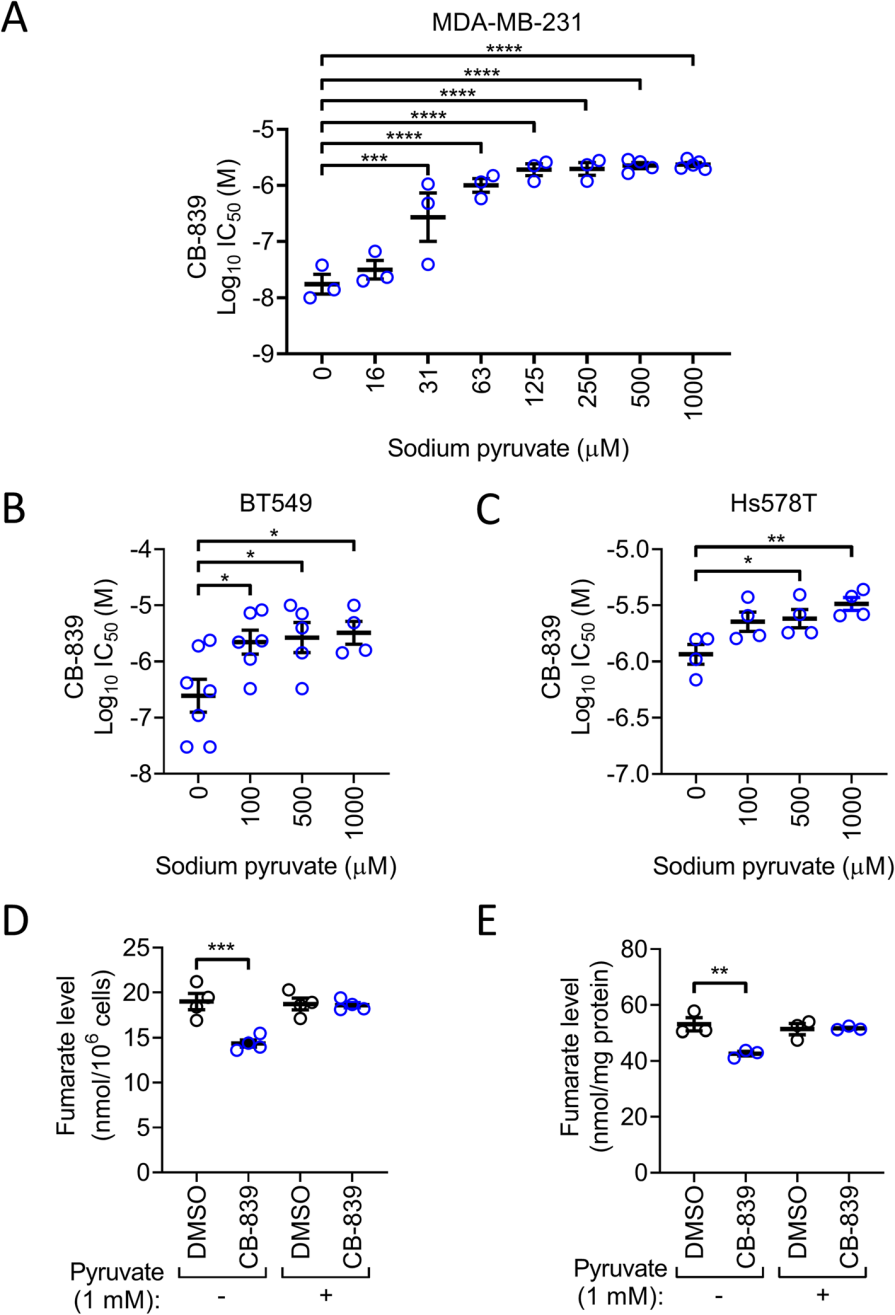
Pyruvate impairs sensitivity to glutaminase inhibition by increasing TCA cycle anaplerosis. Increasing sodium pyruvate concentration in RPMI 1640 + 5% FBS increases CB-839 IC_50_ (mean ± SEM, *n* = 3–7, one-way ANOVA) in **a** MDA-MB-231, **b** BT549 or **c** Hs578T cells during a 3 day assay. 24 h treatment with CB-839 decreased fumarate level in MDA-MB-231 cells in RPMI 1640 + 5% FBS but not RPMI 1640 + 5% FBS supplemented with 1 mM sodium pyruvate. Fumarate level was normalised to either initial cell number seeded (**d**) or protein amount at endpoint (**e**) (mean ± SEM, n = 3–4, two-way ANOVA)

To test this hypothesis MDA-MB-231 cultures were treated with CB-839 in RPMI 1640 supplemented with increasing concentrations of sodium pyruvate (Fig. 2a). The introduction of sodium pyruvate resulted in a concentration-dependent increase in the CB-839 IC_50_ value from 20.7 ± 8.7 nM (*n* = 3) in the unsupplemented RPMI 1640 to 2.4 ± 0.2 μM (*n* = 5) in the culture medium supplemented with 1 mM sodium pyruvate (mean ± sem). All pyruvate concentrations above 31 μM resulted in statistically significant increases in CB-839 IC_50_ compared with unsupplemented RPMI 1640.

BT549 and Hs578T also demonstrated pyruvate concentration-dependent changes in glutaminase inhibitor sensitivity (Fig. 2b and c). In BT549 cultures CB-839 IC_50_ increased from 0.74 ± 0.37 μM (*n* = 7) in the unsupplemented culture medium to 4.5 ± 2.0 μM (*n* = 4) in the culture medium supplemented with 1 mM sodium pyruvate (mean ± sem). CB-839 IC_50_ was significantly increased at pyruvate concentrations ≥100 μM. In Hs578T cultures CB-839 IC_50_ increased from 1.2 ± 0.2 μM in the unsupplemented culture medium to 3.3 ± 0.4 μM in the culture medium supplemented with 1 mM sodium pyruvate (*n* = 4). In this cell line the CB-839 IC_50_ was significantly increased at pyruvate concentrations ≥500 μM.

In sensitive cells glutaminase inhibition using CB-839 was reported to decrease the concentration of many TCA cycle intermediates, including malate, citrate and fumarate [6, 23]. To determine whether exogenous pyruvate can restore TCA cycle intermediates during glutaminase inhibition we assessed steady-state fumarate levels, as a measure of TCA cycle anaplerosis. Cellular fumarate levels were significantly decreased following CB-839 treatment (Fig. 2d and e), in agreement with published findings [6]. Addition of 1 mM sodium pyruvate to RPMI 1640 culture medium prevented this decrease, suggesting that exogenous pyruvate can act as a TCA cycle anaplerotic substrate in TNBC cells when glutamine metabolism is pharmacologically inhibited.

Further studies were conducted to determine whether these effects were dependent to pyruvate carboxylase (PC) activity (Supplementary Figure S1). Treatment with phenylacetic acid (PAA) to inhibit PC was unable to sensitise MDA-MB-231, Hs578T and MCF7 cells to CB-839 in pyruvate-containing culture medium, with a small effect observed in BT549 cells. This finding suggests that in these cell line models replenishment of TCA cycle intermediates by pyruvate during conditions of glutaminase inhibition is largely dependent on the pyruvate dehydrogenase complex and not on PC activity.

The expression of 12 genes involved in pyruvate uptake, mitochondrial transport and metabolism was compared for the 11 breast cancer cell lines using data from the Cancer Cell Line Encyclopedia [30]. The cell lines that did not respond to exogenous pyruvate (T47D and BT474) expressed low levels of MCT genes (Supplementary Table S2). In contrast, the other cell lines that did respond to extracellular pyruvate expressed high levels of either *SLC16A1* (MCT1) or *SLC16A3* (MCT4). This suggests that MCT expression may be a key determinant that allows extracellular pyruvate to suppress CB-839 response.

### Production and paracrine secretion of pyruvate by TNBC cells impairs CB-839 potency

**Fig. 3:**
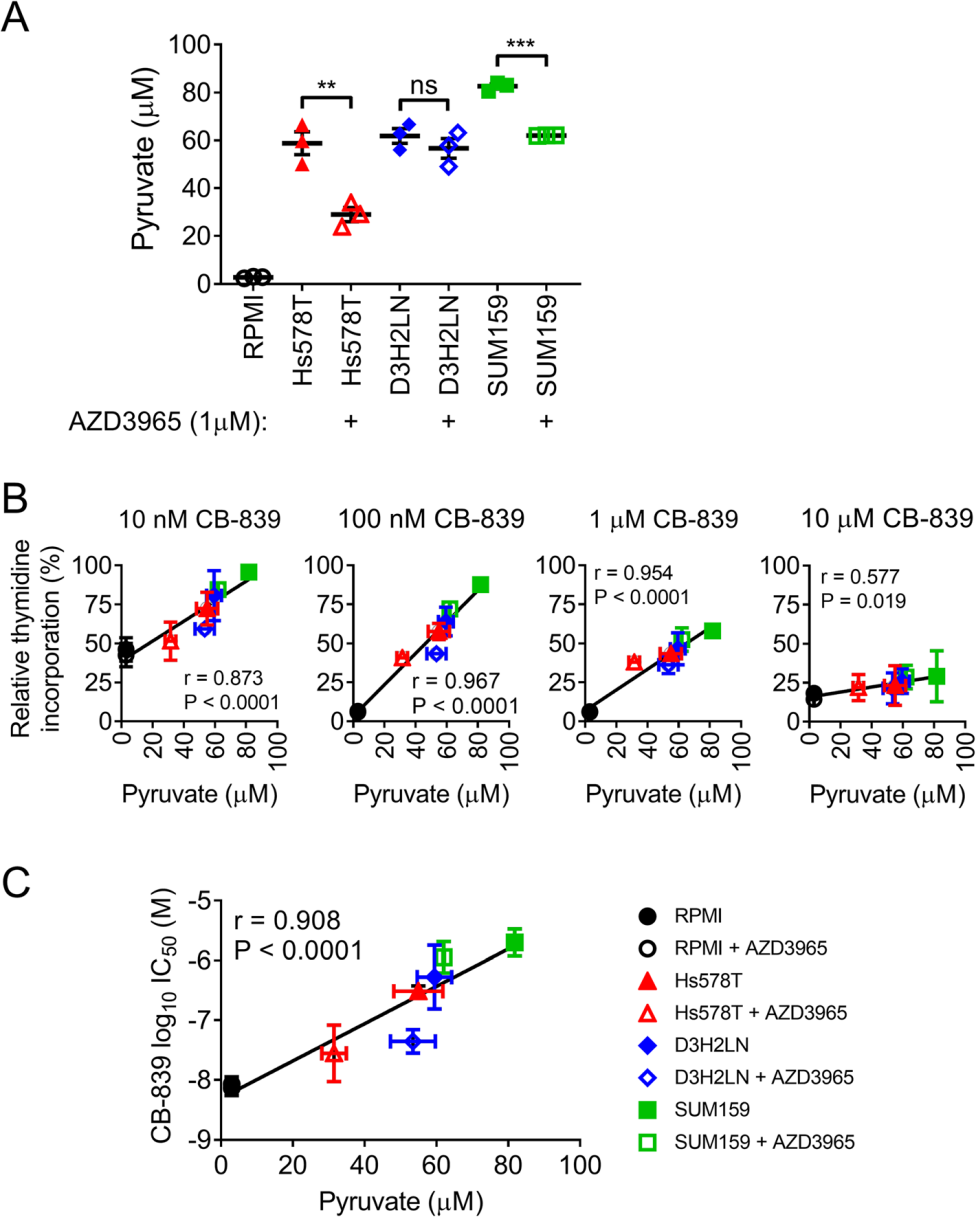
Pyruvate secreted by TNBC cell lines reduces the potency of CB-839. **a** Pharmacological MCT1 inhibition using 1 μM AZD3965 reduced the secretion of pyruvate by Hs578T and SUM159PT (SUM159) but not MDA-MB-231-luc-D3H2LN (D3H2LN) cells. Pyruvate concentration in the conditioned or unconditioned RPMI 1640 + 5% FBS (RPMI) culture medium was quantified after 48 h incubation (mean ± SEM, *n* = 3, t test). **b** The pyruvate concentration in the conditioned culture medium from **a** correlates with resistance of recipient MDA-MB-231 cells to CB-839-treatment at 10 nM, 100 nM, 1 μM or 10 μM over 3 days exposure. For each of the TNBC cell lines studied the AZD3965-treated samples of conditioned culture medium demonstrated a decrease in relative thymidine incorporation in recipient MDA-MB-231 cells (mean ± SD, *n* = 2). **c** IC_50_ analysis also demonstrates a correlation between CB-839 sensitivity and pyruvate concentration in the conditioned culture medium from **a** (mean ± SD, *n* = 2). Correlations were computed by Pearson r correlation coefficient analysis

The pyruvate concentration in RPMI 1640 + 5% FBS culture medium was quantified at 2.8 ± 0.2 μM (mean ± sem, *n* = 3, Fig. 3a). As RPMI 1640 does not contain sodium pyruvate the pyruvate detected likely comes from the 5% (v/v) serum, in line with an approximate concentration of 50–70 μM in the undiluted FBS. We compared pyruvate secretion by three cell lines Hs578T, SUM159PT and a metastatic variant of MDA-MB-231 (MDA-MB-231-luc-D3H2LN). After 48 h culture the resulting pyruvate concentrations in the conditioned culture medium were 58.8 ± 4.8, 61.9 ± 3.1 and 83.9 ± 1.0 μM for Hs578T, MDA-MB-231-luc-D3H2LN and SUM159PT cells, respectively (Fig. 3a). When treated with the MCT1 inhibitor AZD3965 at 1 μM the concentration of pyruvate in the conditioned culture medium was decreased to 28.9 ± 2.9, 56.7 ± 4.1 and 62.1 ± 0.1 μM for Hs578T, MDA-MB-231-luc-D3H2LN and SUM159PT cells, respectively (Fig. 3a). Thus, pharmacological MCT1 inhibition can decrease the secretion of pyruvate by TNBC cells into the extracellular environment.

These samples of conditioned (or unconditioned) culture medium were then supplemented with various concentrations of CB-839 and used to culture drug-naïve MDA-MB-231 cells. The conditioned culture medium with the highest concentrations of pyruvate provided resistance to CB-839, in agreement with studies using sodium pyruvate supplementation (Fig. 3b and c). Indeed, strong linear correlations were observed between the relative % of ^3^H-thymidine incorporation and pyruvate concentration in the samples supplemented with CB-839 at all four concentrations, suggesting that higher levels of pyruvate impair CB-839 activity (Fig. 3b). Similarly, when CB-839 IC_50_ was calculated a strong linear correlation was observed between CB-839 IC_50_ and pyruvate concentration, also supporting the hypothesis that higher extracellular levels of pyruvate impair CB-839 activity (Fig. 3c). Notably, the AZD3965-treated samples from all three cell lines (Hs578T, MDA-MB-231-luc-D3H2LN and SUM159PT) provided lower CB-839 IC_50_ in recipient MDA-MB-231 cells, confirming that MCT1 inhibition can increase CB-839 sensitivity in this in vitro setting.

### TNBC cells grown as 3D spheroids display reduced sensitivity to CB-839 compared with 2D cultures

**Fig. 4:**
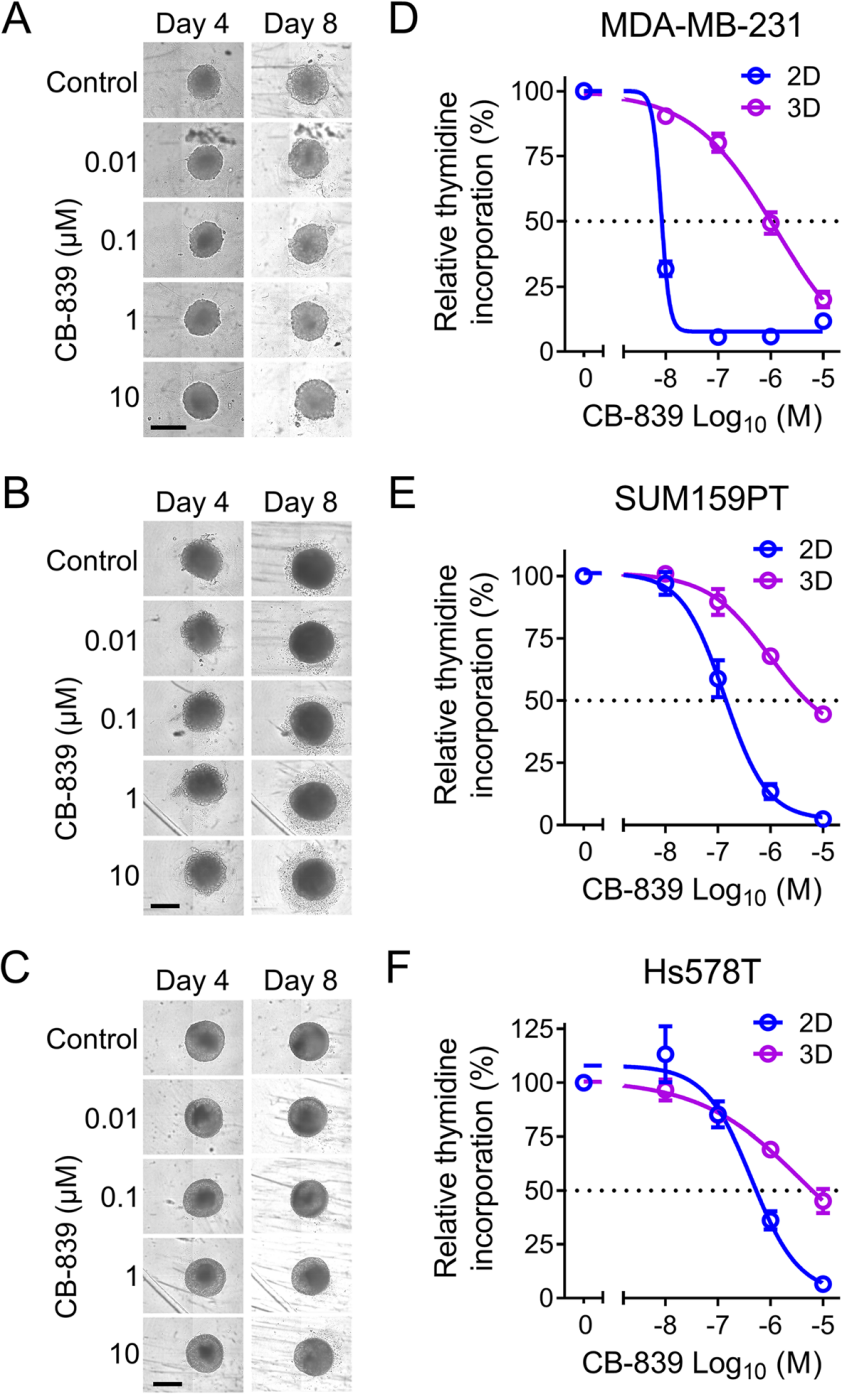
Activity of CB-839 in 3D spheroid versus 2D monolayer cell cultures. Images of MDA-MB-231 (**a**), SUM159PT (**b**) and Hs578T (**c**) 3D spheroid cell cultures captured on day 4 and 8 (scale bar = 0.5 mm). Thymidine incorporation in MDA-MB-231 (**d**), SUM159PT (**e**) and Hs578T (**f**) 3D spheroid cultures and 2D monolayer cultures following 4 days treatment with CB-839 (mean ± SEM, *n* = 4–6)

To investigate the possibility of impaired CB-839 activity due to paracrine environmental effects we used an in vitro 3D spheroid culture model. Following 4 days establishment spheroid cultures were treated with 0.01, 0.1, 1 or 10 μM CB-839. MDA-MB-231 and SUM159PT formed regular slow-growing 3D structures (Fig. 4a and b). Hs578T formed regular spherical clusters that displayed minimal increase in size from day 4 to day 8 (Fig. 4c). Microscopic imaging demonstrated minimal effects of CB-839-treatment on spheroid growth/integrity, even after 4 days exposure to 10 μM of drug. However, when cell proliferation in these cultures was assessed using ^3^H-thymidine incorporation (16 h overnight incubation from day 7 to day 8), CB-839 caused a clear concentration-dependent decrease in cell proliferation in all three cell lines (Fig. 4d-f). In MDA-MB-231 spheroids the CB-839 concentration needed to halve the relative amount of thymidine incorporation (i.e. IC_50_) was 0.88 ± 0.26 μM. This change represents a loss in sensitivity of > 100-fold compared with the 2D monolayer IC_50_ of 8.4 ± 0.17 nM (*n* = 4, *P* = 0.015, t test). This 2D IC_50_ for a 4 day CB-839 exposure was slightly lower than the 19.3 and 20.7 nM IC_50_ values previously observed with 3 day drug exposure (Fig. 1a and 2a).

A 32-fold loss of CB-839 activity was observed in SUM159PT cells when they were grown as 3D spheroids. The IC_50_ of CB-839 was 5.0 ± 0.96 μM for 3D cultures compared with 0.15 ± 0.041 μM for 2D monolayers (*n* = 4, *P* = 0.003). Similarly, a 14-fold loss of CB-839 activity was observed in Hs578T with 3D IC_50_ of 6.6 ± 2.0 μM compared with 2D IC_50_ 0.45 ± 0.022 μM (*n* = 4, *P* = 0.019). Again, the 4 day drug exposure produced a modestly lower IC_50_ than the 3 day IC_50_ of 1.0 and 1.2 μM observed previously (Figs. 1b and 2c). Thus, in the three TNBC cell lines studied CB-839 sensitivity was significantly impaired when cells were grown as 3D spheroids.

The plasma AUC (0–8 h) in patients treated with CB-839 in a 600 mg BID (fed) schedule was approximately 11 μM*h with C_max_ of 2.3 μM and C_min_ of 0.65 μM [31]. Thus, continuous treatment of the spheroids with CB-839 at 1 μM represents an approximate exposure which may be achievable in patient tumours. At this concentration there was a substantial decrease in CB-839 sensitivity in all of the spheroid models tested compared with regular 2D cell culture conditions.

## Discussion

Human studies using isotope-labelled glucose or glutamine are providing a clearer understanding of the metabolic processes, and heterogeneity therein, that occur in tumours. Whilst lung and brain cancers appear to fuel the TCA cycle *via* pyruvate dehydrogenase complex-dependent glucose metabolism, the results from studies in clear cell renal cell carcinoma (ccRCC) display supressed glucose oxidation in the TCA cycle, more reflective of the classic “Warburg Effect” observed in most in vitro studies [17, 32]. In agreement, ^1-13^C-glutamine studies in VHL-deficient ccRCC tumour xenografts confirm that glutamine is a significant anaplerotic nutrient [33]. Other in vitro isotope-labelled tracer findings highlight the requirement for glutamine anaplerosis in conditions of glucose deprivation, a common occurrence in the microenvironment of solid tumours [7]. Notably, isotope-labelled tracer studies have not yet been reported for human TNBC. However, untargeted mass spectrometry-based profiling suggests that glutamine utilisation (glutamate/glutamine ratio) is increased in oestrogen receptor (ER)-negative tumours, at least when compared with ER-positive tumours [15, 34]. Further studies are needed to determine the extent, heterogeneity and products of glutamine metabolism in human TNBC.

The anaplerotic role of glutamine can be bypassed to allow cell survival in glutamine-deprived conditions by utilisation of alternative sources of αKG to replenish levels of TCA cycle intermediates, for example, exogenously acquired glutamate. This process suggests the potential for a common mechanism of resistance to pharmacological glutaminase inhibition; replenishment of TCA cycle intermediates using alternative anaplerotic substrates. Mechanistically this effect may decrease the bioenergetic stress following glutaminase inhibition and allow more of the decreased available pool of glutamate to be utilised for glutathione biosynthesis (and cystine import) and thus provide greater tolerance to oxidative stress [35].

In preclinical tumour models of pancreatic ductal adenocarcinoma glutaminase inhibition effectively targeted proliferating tumour cells, but was ineffective against the hypoxic subpopulation of cells [22]. The residual tumours following treatment with glutaminase inhibitor displayed metabolic changes including increased glycolysis and glycogenesis, suggestive of adaptive metabolic reprogramming that compromises therapy efficacy. Thus, diverse mechanisms of resistance are emerging as possible means for cancer cells to escape the effects of glutaminase inhibition. Which of these mechanisms occur and are relevant in the response of human tumours to glutaminase inhibition is yet to be definitively established.

**Fig. 5:**
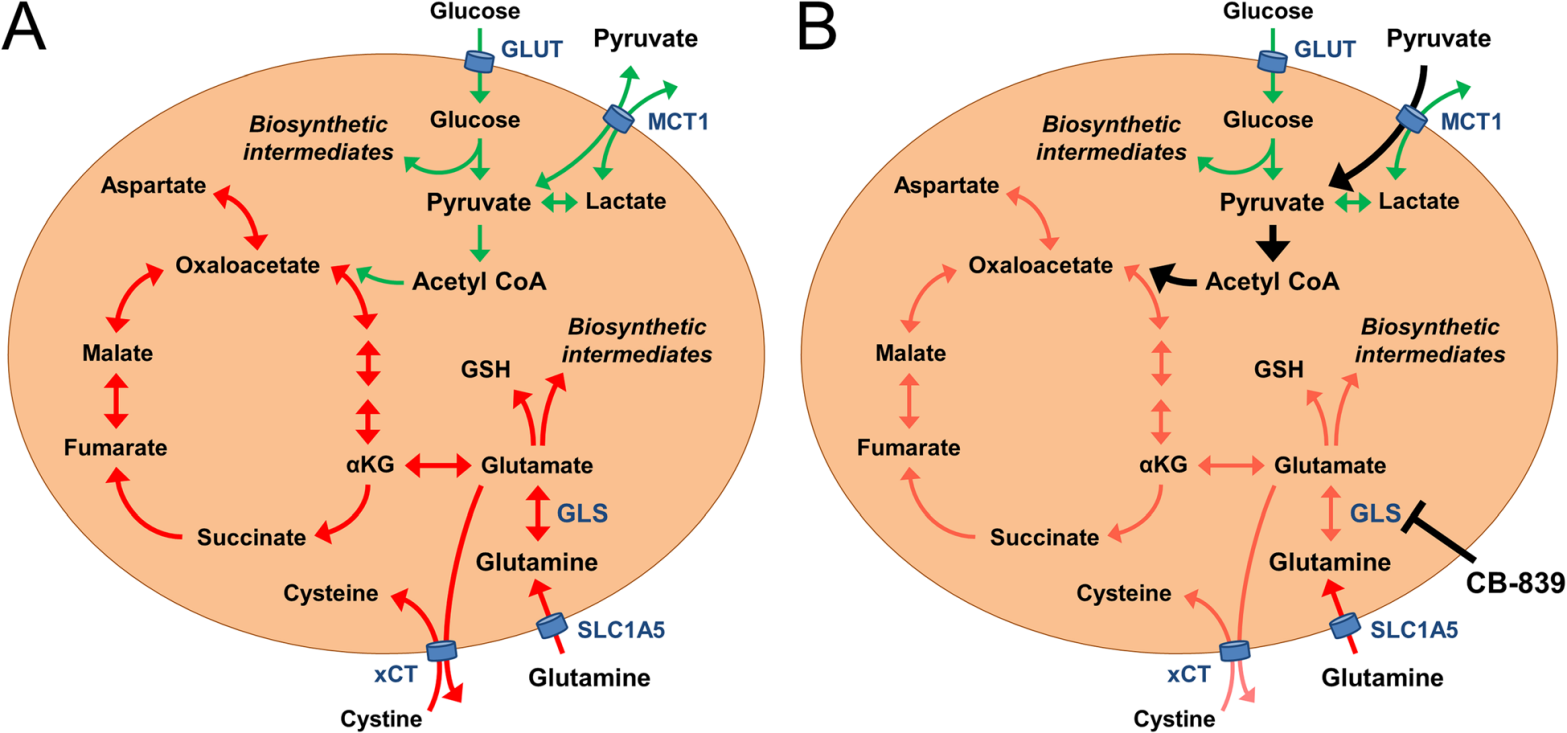
**a** Schematic highlighting metabolic preferences employed by many TNBC cells (glutamine metabolism and related processes in red arrows, glucose metabolism and related processes in green arrows). **b** Schematic demonstrating suppression of glutamine metabolism following glutaminase inhibition by CB-839. Extracellular pyruvate either from systemic circulation or paracrine supply can be transported into these cells to replenish TCA cycle intermediates and decrease the activity of glutaminase inhibitor. Abbreviations: GLS = glutaminase, GLUT = glucose transporter, GSH = reduced glutathione, MCT1 = monocarboxylate transporter 1, SLC1A5 = glutamine transporter, xCT = glutamate/cystine antiporter. Image created using Microsoft® PowerPoint® 2016 (version 16.0.4266.1001)

Enhanced glutaminolysis is a common feature of many TNBC cell lines that supports cell growth both in vitro and in vivo [36]. Glutamine serves many of the biosynthetic, bioenergetic, epigenetic and redox needs of these cells (Fig. 5a). In this study we demonstrate that the sensitivity of TNBC cell lines to glutaminase inhibition is decreased in culture medium containing pyruvate supplementation. This exogenously added pyruvate was able to rescue levels of the TCA cycle intermediate fumarate in CB-839-treated conditions (Fig. 5b). Furthermore, we examined the levels of endogenously produced pyruvate secreted by TNBC cell lines in culture and show that paracrine supply of pyruvate can significantly reduce the sensitivity of recipient cells to glutaminase inhibition. Of note, our IC_50_ studies are conducted at low cell density (1–3 × 10^3^ cells/well of a 96-well plate in 0.12 mL of culture medium), whereas the pyruvate secretion studies were done at approximately 7.5-fold higher cell:culture medium ratio (1.5–2.5 × 10^5^ cells/well of a 6-well plate in 2 mL of culture medium). Thus, at low cell density/high culture medium volume there will be minimal opportunity for an autocrine/paracrine effect of pyruvate to impair glutaminase inhibitor activity. This insight highlights the importance of using higher cell density 2D and 3D assays in future studies of metabolism-targeted drugs. More broadly, it also argues for the development and detailed characterisation of new cell culture technologies that closely recapitulate the in vivo tumour microenvironment.

Whist the focus of this study is on pyruvate, it is also possible that lactate might also act in a similar manner. Lactate concentrations are elevated in many cancer types, including breast [37]. Incorporation of ^13^C-lactate into TCA cycle intermediates is extensive, suggesting a major role for lactate as an oxidative fuel [38]. Considering the similarities in pyruvate and lactate transport (by the same MCT transporters) and potential for interconversion catalysed by lactate dehydrogenase (LDH) enzymes, future studies should consider the possible role of lactate in CB-839 responsiveness.

TNBC cells are known to produce and secrete glutamate [39]. This extracellular glutamate (at moderate concentrations) may play an anaplerotic role during conditions of glutamine deprivation. However, glutamate secretion is required for cystine uptake *via* the xCT antiporter system and excess accumulation in the extracellular compartment can impair xCT function and limit cystine uptake – a process that is required to support glutaminolysis [19, 39]. Thus, excessive extracellular glutamate levels may also act in the acquisition of glutamine independence in these models.

To our knowledge this is the first reported usage of 3D spheroid cell culture models as a tool to study the pharmacology of glutaminase inhibitors. Our findings demonstrate that the potency of glutaminase inhibition is impaired in 3D spheroid models compared with the 2D monolayer cultures commonly used for evaluating drug activity. There are a number of potential reasons for this change. The proportion of proliferating cells in 3D models is lower than in 2D cultures resulting in greater cellular heterogeneity within the culture [40, 41]. The altered nutrient composition, oxygen deprivation and cellular responses including activity of growth signalling cascades and amino acid/hypoxic stress responses may influence glutamine preference. Unequal drug penetration and distribution of CB-839 throughout the spheroid may also impair efficacy compared with 2D cultures.

Multicellular spheroids represent useful models of many of the microenvironmental characteristics that occur in tumours. Advanced mathematical modelling of these systems can provide key insights into cell proliferation/death, oxygen and nutrient gradients, development of necrosis, and how these compartments interact and change spatially and temporally with spheroid growth and in response to treatment [42]. It has been reported that the anchorage-independent growth conditions of spheroid culture alter glutamine metabolism, favouring reductive carboxylation of glutamine to citrate to support redox homeostasis [43]. Glucose metabolism was also altered in 3D compared with 2D cultures, with lower abundance of glucose-derived citrate suggestive of decreased pyruvate dehydrogenase activity [43]. Whether these effects occur in TNBC spheroid models is yet to be determined.

Another key advantage of 3D spheroids is their relative ease of use. They can be established rapidly (within a few days), grown in 96-well format to enable high-throughput studies, manipulated using conventional multichannel dispensing methods, imaged regularly to monitor growth and treatment effects and can be histologically processed at the study endpoint for more detailed microscopic analysis. Whilst 3D cell culture systems, including cell line-derived spheroids and ex vivo patient-derived organoids, are better models of solid tumour pathophysiology, there is still much work needed to characterise their metabolic alterations (using metabolomic techniques) and define appropriate culture medium compositions to model in vivo conditions. Nonetheless, cell line-derived spheroids, in their current form, represent a useful and cost-effective tool for studying inhibitors of cellular metabolism and testing how they can be combined with other therapies prior to investigations using preclinical tumour models and human subjects.

## Conclusion

Cancer cells grown in vitro display different levels of sensitivity to glutaminase inhibition depending on culture medium composition. A single metabolite, pyruvate, was particularly effective in suppressing the potency of glutaminase inhibitor by contributing to TCA cycle anaplerosis. We build upon this mechanistic observation by showing that TNBC cells themselves readily secrete pyruvate into the extracellular environment. This endogenously-derived pyruvate acted in a paracrine manner to decrease the sensitivity of drug-naïve recipient cells to subsequent treatment with CB-839. Blocking this pyruvate secretion, using an MCT1 inhibitor, prevented this process and improved CB-839 activity in the recipient cells. CB-839 potency was also significantly compromised in 3D spheroid compared with 2D monolayer culture models. These new discoveries highlight the potential influence that circulating and tumour-derived pyruvate and other microenvironmental features can have on glutaminase inhibitor efficacy. Future clinical studies to clearly define the metabolomic landscapes of human tumours will help to define which cancer types and tumour features are predictive of response to these agents.

## Supplementary information

**Additional file 1: Supplementary Table S1.** Comparison of RPMI 1640, αMEM and DMEM culture medium formulation.

**Additional file 2: Supplementary Table S2.** mRNA expression data (RNAseq; RMA, log_2_) was downloaded from the Cancer Cell Line Encyclopedia for genes involved in pyruvate uptake (*SLC16A1*, *SLC16A7*, *SLC16A3*, *SLC16A8*, *SLC16A4*), mitochondrial transport (*MPC1*, *MPC2*) and metabolism (*PC*, *PDHA1*, *DLAT*, *PDHX*, *LDHA*) for the 11 breast cancer cell lines used in our study. Cell lines that display increased CB-839 IC_50_ in the presence of pyruvate and associated *P*-value are also presented.

**Additional file 3: Supplementary Figure S1.** Inhibition of Pyruvate Carboxylase using Phenylacetic acid (PAA) does not increase sensitivity to CB-839 in three of four breast cancer cell lines. Cultures were exposed to a titration of CB-839 concentrations in pyruvate-containing αMEM + 5% FBS with or without 5 mM PAA (Sigma-Aldrich). ^3^H-thymidine incorporation was assayed on day 3 and % signal plotted relative to CB-839-untreated samples. MDA-MB-231 (**A**), MCF7 (**B**) and Hs578T (**C**) did not display any increase in CB-839 sensitivity in the presence of PAA (+PAA). In contrast, BT549 (**D**) did demonstrate a slight decrease in the CB-839 IC_50_ in the presence of PAA (mean ± SD, *n* = 4, unpaired t test).

## Data Availability

The datasets during and/or analysed during the current study available from the corresponding author on reasonable request.

## Abbreviations

2D: Two dimensional
3D: Three dimensional
αKG: α-ketoglutarate
αMEM: Minimum Essential Medium α modification
ANOVA: Analysis of variance
ccRCC: Clear cell renal cell carcinoma
DMEM: Dulbecco’s Modified Eagle Medium
DMSO: Dimethyl sulfoxide
ER: Oestrogen receptor
FBS: Foetal bovine serum
GLS: Glutaminase
LDH: Lactate dehydrogenase
MCT: Monocarboxylate transporter
NSCLC: Non-small cell lung cancer
ORR: Objective response rate
PC: Pyruvate carboxylase
RPMI 1640: Roswell Park Memorial Institute 1640 medium
TCA: Tricarboxylic acid
TNBC: Triple receptor-Negative Breast Cancer
xCT: Glutamate/cystine antiporter

## References

[1] Altman BJ, Stine ZE, Dang CV (2016). From Krebs to clinic: glutamine metabolism to cancer therapy. Nat Rev Cancer.

[2] Pavlova NN, Thompson CB (2016). The emerging hallmarks of Cancer metabolism. Cell Metab.

[3] Zhang J, Pavlova NN, Thompson CB (2017). Cancer cell metabolism: the essential role of the nonessential amino acid, glutamine. EMBO J.

[4] Still ER, Yuneva MO (2017). Hopefully devoted to Q: targeting glutamine addiction in cancer. Br J Cancer.

[5] Timmerman LA, Holton T, Yuneva M, Louie RJ, Padro M, Daemen A, Hu M, Chan DA (2013). Ethier SP, van 't veer LJ *et al*: glutamine sensitivity analysis identifies the xCT antiporter as a common triple-negative breast tumor therapeutic target. Cancer Cell.

[6] Gross MI, Demo SD, Dennison JB, Chen L, Chernov-Rogan T, Goyal B, Janes JR, Laidig GJ, Lewis ER, Li J (2014). Antitumor activity of the glutaminase inhibitor CB-839 in triple-negative breast cancer. Mol Cancer Ther.

[7] Le A, Lane AN, Hamaker M, Bose S, Gouw A, Barbi J, Tsukamoto T, Rojas CJ, Slusher BS, Zhang H (2012). Glucose-independent glutamine metabolism via TCA cycling for proliferation and survival in B cells. Cell Metab.

[8] Sun RC, Denko NC (2014). Hypoxic regulation of glutamine metabolism through HIF1 and SIAH2 supports lipid synthesis that is necessary for tumor growth. Cell Metab.

[9] Mullen AR, Wheaton WW, Jin ES, Chen PH, Sullivan LB, Cheng T, Yang Y, Linehan WM, Chandel NS, DeBerardinis RJ (2011). Reductive carboxylation supports growth in tumour cells with defective mitochondria. Nature.

[10] Metallo CM, Gameiro PA, Bell EL, Mattaini KR, Yang J, Hiller K, Jewell CM, Johnson ZR, Irvine DJ, Guarente L (2011). Reductive glutamine metabolism by IDH1 mediates lipogenesis under hypoxia. Nature.

[11] Wise DR, Ward PS, Shay JE, Cross JR, Gruber JJ, Sachdeva UM, Platt JM, DeMatteo RG, Simon MC, Thompson CB (2011). Hypoxia promotes isocitrate dehydrogenase-dependent carboxylation of alpha-ketoglutarate to citrate to support cell growth and viability. Proc Natl Acad Sci U S A.

[12] Huang Q, Stalnecker C, Zhang C, McDermott LA, Iyer P, O'Neill J, Reimer S, Cerione RA, Katt WP (2018). Characterization of the interactions of potent allosteric inhibitors with glutaminase C, a key enzyme in cancer cell glutamine metabolism. J Biol Chem.

[13] McDermott LA, Iyer P, Vernetti L, Rimer S, Sun J, Boby M, Yang T, Fioravanti M, O'Neill J, Wang L (2016). Design and evaluation of novel glutaminase inhibitors. Bioorg Med Chem.

[14] Vidal G, Kalinsky K, Stringer-Reasor E, Lynce F, Cole J, Valdes-Albini F, Soliman H, Nikolinakos P, Silber A, DeMichele A (2019). Abstract P6–20-07: Efficacy and safety of CB-839, a small molecule inhibitor of glutaminase, in combination with paclitaxel in patients with advanced triple negative breast cancer (TNBC): Initial findings from a multicenter, open-label phase 2 study.

[15] Kalinsky K, Harding J, DeMichele A, Infante J, Gogineni K, Owonikoko T, Isakoff S, Iliopoulos O, Patel M, Munster P (2018). Abstract PD3–13: Phase 1 study of CB-839, a first-in-class oral inhibitor of glutaminase, in combination with paclitaxel in patients with advanced triple negative breast cancer.

[16] Davidson SM, Papagiannakopoulos T, Olenchock BA, Heyman JE, Keibler MA, Luengo A, Bauer MR, Jha AK, O'Brien JP, Pierce KA (2016). Environment impacts the metabolic dependencies of Ras-driven non-small cell lung Cancer. Cell Metab.

[17] Hensley CT, Faubert B, Yuan Q, Lev-Cohain N, Jin E, Kim J, Jiang L, Ko B, Skelton R, Loudat L (2016). Metabolic heterogeneity in human lung tumors. Cell.

[18] Sellers K, Fox MP, Bousamra M, Slone SP, Higashi RM, Miller DM, Wang Y, Yan J, Yuneva MO, Deshpande R (2015). Pyruvate carboxylase is critical for non-small-cell lung cancer proliferation. J Clin Invest.

[19] Muir A, Danai LV, Gui DY, Waingarten CY, Lewis CA, Vander Heiden MG. Environmental cystine drives glutamine anaplerosis and sensitizes cancer cells to glutaminase inhibition. eLife. 2017;6:e27713. Published 2017 Aug 15. 10.7554/eLife.27713. 10.7554/eLife.27713 PMC5589418 28826492

[20] Sayin VI, LeBoeuf SE, Singh SX, Davidson SM, Biancur D, Guzelhan BS, Alvarez SW, Wu WL, Karakousi TR, Zavitsanou AM, et al. Activation of the NRF2 antioxidant program generates an imbalance in central carbon metabolism in cancer. eLife. 2017;6:e28083. Published 2017 Oct 2. 10.7554/eLife.28083. 10.7554/eLife.28083 PMC5624783 28967864

[21] Son J, Lyssiotis CA, Ying H, Wang X, Hua S, Ligorio M, Perera RM, Ferrone CR, Mullarky E, Shyh-Chang N (2013). Glutamine supports pancreatic cancer growth through a KRAS-regulated metabolic pathway. Nature.

[22] Elgogary A, Xu Q, Poore B, Alt J, Zimmermann SC, Zhao L, Fu J, Chen B, Xia S, Liu Y (2016). Combination therapy with BPTES nanoparticles and metformin targets the metabolic heterogeneity of pancreatic cancer. Proc Natl Acad Sci U S A.

[23] Biancur DE, Paulo JA, Malachowska B, Quiles Del Rey M, Sousa CM, Wang X, Sohn ASW, Chu GC, Gygi SP, Harper JW (2017). Compensatory metabolic networks in pancreatic cancers upon perturbation of glutamine metabolism. Nat Commun.

[24] Yuneva M, Zamboni N, Oefner P, Sachidanandam R, Lazebnik Y (2007). Deficiency in glutamine but not glucose induces MYC-dependent apoptosis in human cells. J Cell Biol.

[25] Patel D, Menon D, Bernfeld E, Mroz V, Kalan S, Loayza D, Foster DA (2016). Aspartate rescues S-phase arrest caused by suppression of glutamine utilization in KRas-driven Cancer cells. J Biol Chem.

[26] Olek RA, Kujach S, Wnuk D, Laskowski R (2014). Single sodium pyruvate ingestion modifies blood acid-base status and post-exercise lactate concentration in humans. Nutrients.

[27] Landon J, Fawcett JK, Wynn V (1962). Blood pyruvate concentration measured by a specific method in control subjects. J Clin Pathol.

[28] Daly ME, Vale C, Walker M, Littlefield A, Alberti KG, Mathers JC (1998). Acute effects on insulin sensitivity and diurnal metabolic profiles of a high-sucrose compared with a high-starch diet. Am J Clin Nutr.

[29] Leung EY, Kim JE, Askarian-Amiri M, Rewcastle GW, Finlay GJ, Baguley BC (2014). Relationships between signaling pathway usage and sensitivity to a pathway inhibitor: examination of trametinib responses in cultured breast cancer lines. PLoS One.

[30] Barretina J, Caponigro G, Stransky N, Venkatesan K, Margolin AA, Kim S, Wilson CJ, Lehar J, Kryukov GV, Sonkin D (2012). The Cancer cell line encyclopedia enables predictive modelling of anticancer drug sensitivity. Nature.

[31] Harding JJ, Telli ML, Munster PN, Le MH, Molineaux C, Bennett MK, Mittra E, Burris HA, Clark AS, Dunphy M (2015). Safety and tolerability of increasing doses of CB-839, a first-in-class, orally administered small molecule inhibitor of glutaminase, in solid tumors.

[32] Courtney KD, Bezwada D, Mashimo T, Pichumani K, Vemireddy V, Funk AM, Wimberly J, McNeil SS, Kapur P, Lotan Y (2018). Isotope tracing of human clear cell renal cell carcinomas demonstrates suppressed glucose oxidation in vivo. Cell Metab.

[33] Gameiro PA, Yang J, Metelo AM, Perez-Carro R, Baker R, Wang Z, Arreola A, Rathmell WK, Olumi A, Lopez-Larrubia P (2013). In vivo HIF-mediated reductive carboxylation is regulated by citrate levels and sensitizes VHL-deficient cells to glutamine deprivation. Cell Metab.

[34] Terunuma A, Putluri N, Mishra P, Mathe EA, Dorsey TH, Yi M, Wallace TA, Issaq HJ, Zhou M, Killian JK (2014). MYC-driven accumulation of 2-hydroxyglutarate is associated with breast cancer prognosis. J Clin Invest.

[35] Daemen A, Liu B, Song K, Kwong M, Gao M, Hong R, Nannini M, Peterson D, Liederer BM, de la Cruz C (2018). Pan-Cancer metabolic signature predicts co-dependency on Glutaminase and De novo glutathione synthesis linked to a high-Mesenchymal cell state. Cell Metab.

[36] Lampa M, Arlt H, He T, Ospina B, Reeves J, Zhang B, Murtie J, Deng G, Barberis C, Hoffmann D (2017). Glutaminase is essential for the growth of triple-negative breast cancer cells with a deregulated glutamine metabolism pathway and its suppression synergizes with mTOR inhibition. PLoS One.

[37] de la Cruz-Lopez KG, Castro-Munoz LJ, Reyes-Hernandez DO, Garcia-Carranca A, Manzo-Merino J (2019). Lactate in the regulation of tumor microenvironment and therapeutic approaches. Front Oncol.

[38] Hui S, Ghergurovich JM, Morscher RJ, Jang C, Teng X, Lu W, Esparza LA, Reya T, Le Z, Yanxiang Guo J (2017). Glucose feeds the TCA cycle via circulating lactate. Nature.

[39] Briggs KJ, Koivunen P, Cao S, Backus KM, Olenchock BA, Patel H, Zhang Q, Signoretti S, Gerfen GJ, Richardson AL (2016). Paracrine induction of HIF by glutamate in breast Cancer: EglN1 senses cysteine. Cell.

[40] Melissaridou S, Wiechec E, Magan M, Jain MV, Chung MK, Farnebo L, Roberg K (2019). The effect of 2D and 3D cell cultures on treatment response, EMT profile and stem cell features in head and neck cancer. Cancer Cell Int.

[41] Riedl A, Schlederer M, Pudelko K, Stadler M, Walter S, Unterleuthner D, Unger C, Kramer N, Hengstschlager M, Kenner L (2017). Comparison of cancer cells in 2D vs 3D culture reveals differences in AKT-mTOR-S6K signaling and drug responses. J Cell Sci.

[42] Mao X, McManaway S, Jaiswal JK, Patel PB, Wilson WR, Hicks KO, Bogle G (2018). An agent-based model for drug-radiation interactions in the tumour microenvironment: hypoxia-activated prodrug SN30000 in multicellular tumour spheroids. PLoS Comput Biol.

[43] Jiang L, Shestov AA, Swain P, Yang C, Parker SJ, Wang QA, Terada LS, Adams ND, McCabe MT, Pietrak B (2016). Reductive carboxylation supports redox homeostasis during anchorage-independent growth. Nature.

