# Supplementary figures and images for: Pyruvate anaplerosis is a mechanism of resistance to pharmacological glutaminase inhibition in triple-receptor negative breast cancer
Source: BMC Cancer. 2020 May 25;20:470. doi: 10.1186/s12885-020-06885-3 (PMC7333265; doi:10.1186/s12885-020-06885-3)

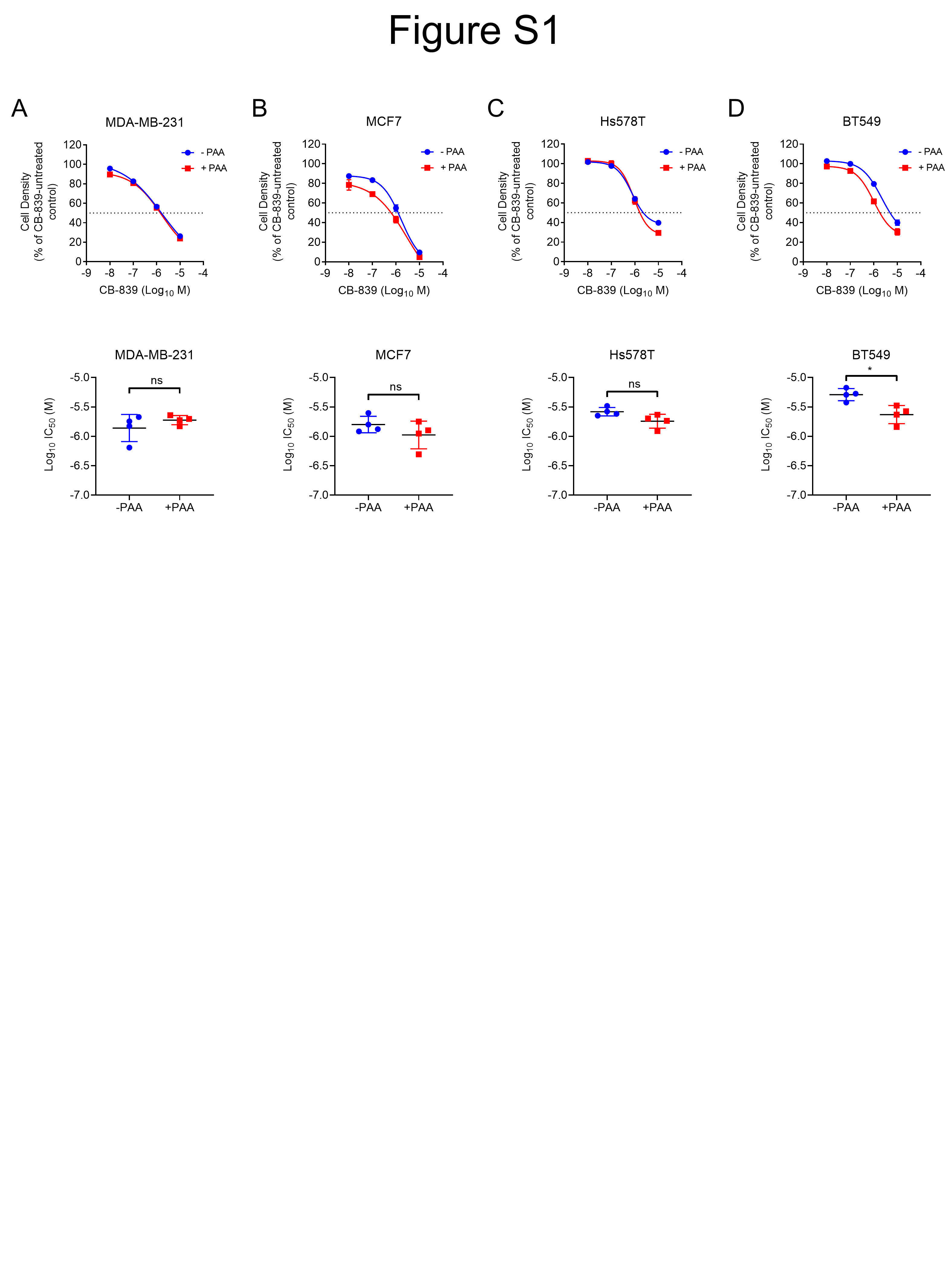

Supplement: Supplementary file 3 — Additional file 3: Supplementary Figure S1. Inhibition of Pyruvate Carboxylase using Phenylacetic acid (PAA) does not increase sensitivity to CB-839 in three of four breast cancer cell lines. Cultures were exposed to a titration of CB-839 concentrations in pyruvate-containing αMEM + 5% FBS with or without 5 mM PAA (Sigma-Aldrich). 3H-thymidine incorporation was assayed on day 3 and % signal plotted relative to CB-839-untreated samples. MDA-MB-231 (A), MCF7 (B) and Hs578T (C) did not display any increase in CB-839 sensitivity in the presence of PAA (+PAA). In contrast, BT549 (D) did demonstrate a slight decrease in the CB-839 IC50 in the presence of PAA (mean ± SD, n = 4, unpaired t test). [file 12885_2020_6885_MOESM3_ESM.tif]
